# Myoepithelioma-Like Tumor of the Vulvar Region Presenting as a Painful Vulvar Nodule in a Young Woman: A Case Report

**DOI:** 10.7759/cureus.114809

**Published:** 2026-08-19

**Authors:** Mitsuaki Yoshida, Thao T Nguyen, Takahiro Iwata, Miyako Shimasaki, Motona Kumagai, Akihiro Shioya, Takeru Oyama, Katuaki Sato, Yojiro Tsuda, Masanori Hisaoka, Sohsuke Yamada

**Affiliations:** 1 Department of Pathology and Laboratory Medicine, Kanazawa Medical University, Uchinada, JPN; 2 Department of Pathology I, Kanazawa Medical University, Uchinada, JPN; 3 Department of Pathology, Kanazawa Medical University Hospital, Uchinada, JPN; 4 School of Medicine, Kanazawa Medical University, Uchinada, JPN; 5 Department of Pathology II, Kanazawa Medical University, Uchinada, JPN; 6 Department of Pathology Ⅱ, Kanazawa Medical University, Uchinada, JPN; 7 Department of Diagnostic Pathology, Noto General Hospital, Nanao, JPN; 8 Department of Pathology and Oncology, University of Occupational and Environmental Health, Kitakyushu, JPN

**Keywords:** case reports, myoepithelioma, smarcb1 protein, soft tissue neoplasms, vulva

## Abstract

Myoepithelioma-like tumor of the vulvar region (MELTVR) is a rare mesenchymal subcutaneous tumor of low malignant potential that occurs in adult women and has typical histological features, including loss of SMARCB1 (INI1) expression. This report describes the case of a 23-year-old woman with a painful vulvar subcutaneous nodule and a diagnosis of MELTVR and describes the challenges in the approach to definitive diagnosis. A 23-year-old woman presented with a three-month history of right pubic pain. Imaging revealed a 25-mm inguinal lesion without metastasis. Biopsy demonstrated spindle and plasmacytoid tumor cells in myxoid and hypercellular areas, suggesting myoepithelioma. Although necrosis and mitotic activity were not evaluable in the biopsy specimen, malignant potential could not be excluded. Complete surgical excision was subsequently performed. The resected tumor measured 45 × 35 × 15 mm and showed multilocular solid and gelatinous areas. Histologically, lobulated growth, marked atypia, necrosis, and mitotic activity (9/10 HPF) were identified. Immunohistochemically, tumor cells showed positivity for estrogen receptor (ER) and progesterone receptor (PgR), along with loss of SMARCB1 (INI1) expression. Other markers, such as neural and myoepithelial markers, were negative. Fluorescence in situ hybridization (FISH) analysis showed no Ewing sarcoma breakpoint region 1 (EWSR1) rearrangement. MELTVR was diagnosed based on integrated clinicopathological and molecular findings. Biopsy specimens may fail to capture key diagnostic features of MELTVR because of intratumoral heterogeneity. Definitive diagnosis requires comprehensive evaluation of resection specimens with immunohistochemical and molecular analyses. This case may contribute to establishing diagnostic criteria for this rare entity.

## Introduction

Myoepithelioma-like tumor of the vulvar region (MELTVR) is a rare SMARCB1-deficient mesenchymal neoplasm arising predominantly in the vulva of adult women [1-5]. Since its initial description by Yoshida et al. in 2015 [1], approximately 20 cases have been reported in the available literature to date. This entity is not included in the current WHO Classification of Soft Tissue and Bone Tumors [1,6]. Histologically, MELTVR is characterized by spindle to epithelioid tumor cells showing biphasic growth in myxoid and cellular areas and, immunohistochemically, by frequent expression of estrogen receptor (ER) and progesterone receptor (PgR), together with loss of SMARCB1 (INI1) expression [1-5,7-10]. Unlike conventional soft tissue myoepithelioma, MELTVR typically demonstrates frequent ER and PgR expression together with loss of SMARCB1 (INI1) expression and arises predominantly in the vulvar region of adult women. Although it shares SMARCB1 deficiency with other tumors, such as epithelioid sarcoma, its characteristic morphology, anatomical predilection, and immunophenotype support its recognition as a distinct clinicopathological entity. Although MELTVR is generally considered a tumor of low malignant potential, local recurrence has been reported in a small number of cases [4]. Surgical excision with negative margins is currently regarded as the treatment of choice [1-5]. Previous reports have also highlighted that definitive diagnosis is often achieved only after examination of resection specimens [3,7].

Therefore, comprehensive histopathological, immunohistochemical, and molecular evaluation is essential for establishing an accurate diagnosis, particularly when biopsy findings are limited. Herein, we report a case of MELTVR presenting as a painful vulvar nodule in a young woman and describe its clinicopathological, immunohistochemical, and molecular features. This report highlights the diagnostic limitations of biopsy specimens and emphasizes the importance of integrated histopathological, immunohistochemical, and molecular evaluation for accurate diagnosis.

## Case presentation

**Figure 1:**
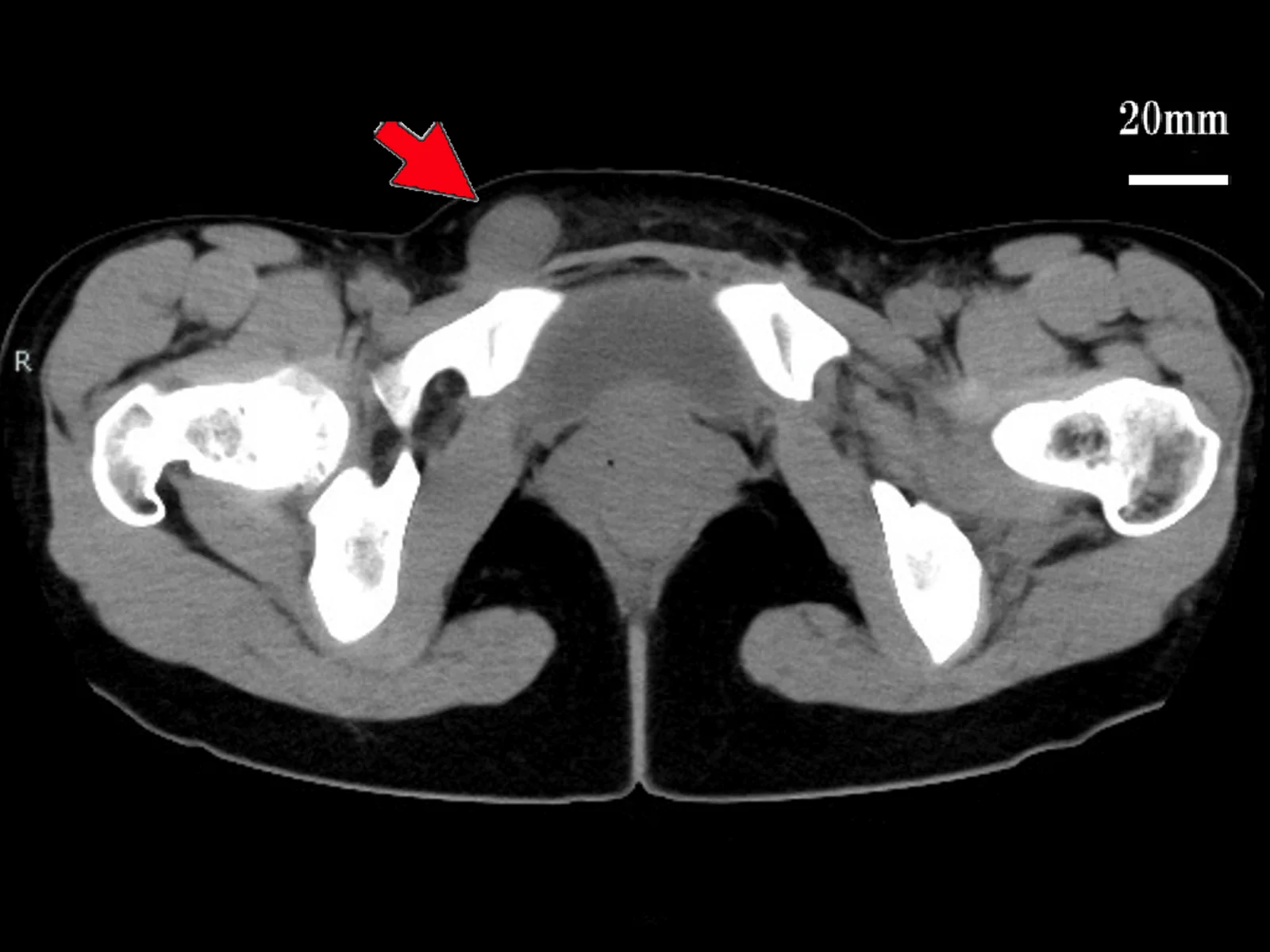
Plain pelvic computed tomography scan. A well-circumscribed 25-mm low-attenuation solid lesion is identified in the right inguinal region (arrow).

A 23-year-old woman presented with a three-month history of right pubic pain. She had a history of two deliveries by cesarean section and no other significant medical or surgical history. She had experienced pain around the right pubic region for three months. Due to persistent discomfort, she visited a previous clinic. Physical examination revealed an approximately 3-cm, well-circumscribed, mobile subcutaneous nodule adjacent to her previous cesarean section scar. Laboratory tests, including complete blood count and serum biochemistry, were unremarkable. Plain computed tomography revealed a 25-mm low-attenuation solid lesion in the right inguinal region. No additional nodules or masses were identified, and there was no radiologic evidence of metastasis (Figure 1). 

**Figure 2:**
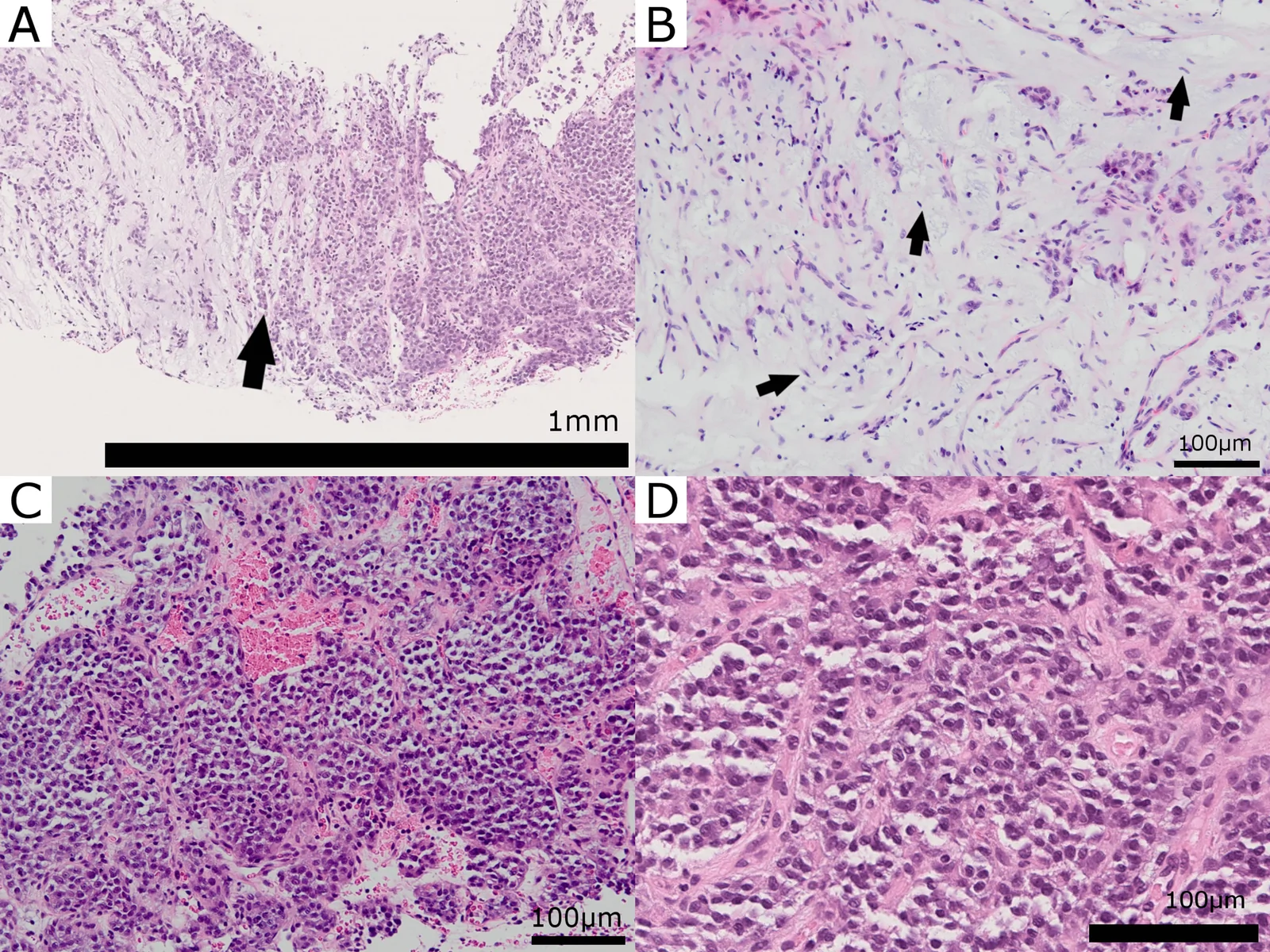
Histological findings of the biopsy specimen. (A) Transition between hypocellular myxoid areas (left) and hypercellular areas (right) (hematoxylin and eosin (H&E) staining, objective magnification ×3). Arrows indicate the transition zone between the hypocellular myxoid and hypercellular areas. (B) Hypocellular myxoid area showing spindle-shaped tumor cells embedded in abundant myxoid stroma (H&E, ×10). Arrows indicate representative spindle-shaped tumor cells. (C, D) Hypercellular areas composed of round to oval plasmacytoid tumor cells with eccentrically located nuclei and hyaline globule-like cytoplasm (C: H&E, ×10; D: H&E, ×40).

A biopsy of the lesion was performed. The biopsy specimen showed transition areas between hypocellular myxoid regions and hypercellular areas (Figure 2A). In the hypocellular myxoid areas, spindle-shaped tumor cells were embedded in abundant myxoid stroma (Figure 2B). In the hypercellular areas, tumor cells had round to oval nuclei with eccentrically located plasmacytoid morphology and hyaline globule-like cytoplasm (Figure 2C, 2D). Necrosis and mitotic figures were not identified in the biopsy specimen. 

Based on the biopsy, the differential diagnoses included soft tissue myoepithelioma, myxoid chondrosarcoma, and deep benign fibrous histiocytoma; however, a malignant tumor could not be excluded. The patient subsequently underwent surgical excision of the vulvar tumor, and the resection specimen was submitted for further evaluation.

**Figure 3:**
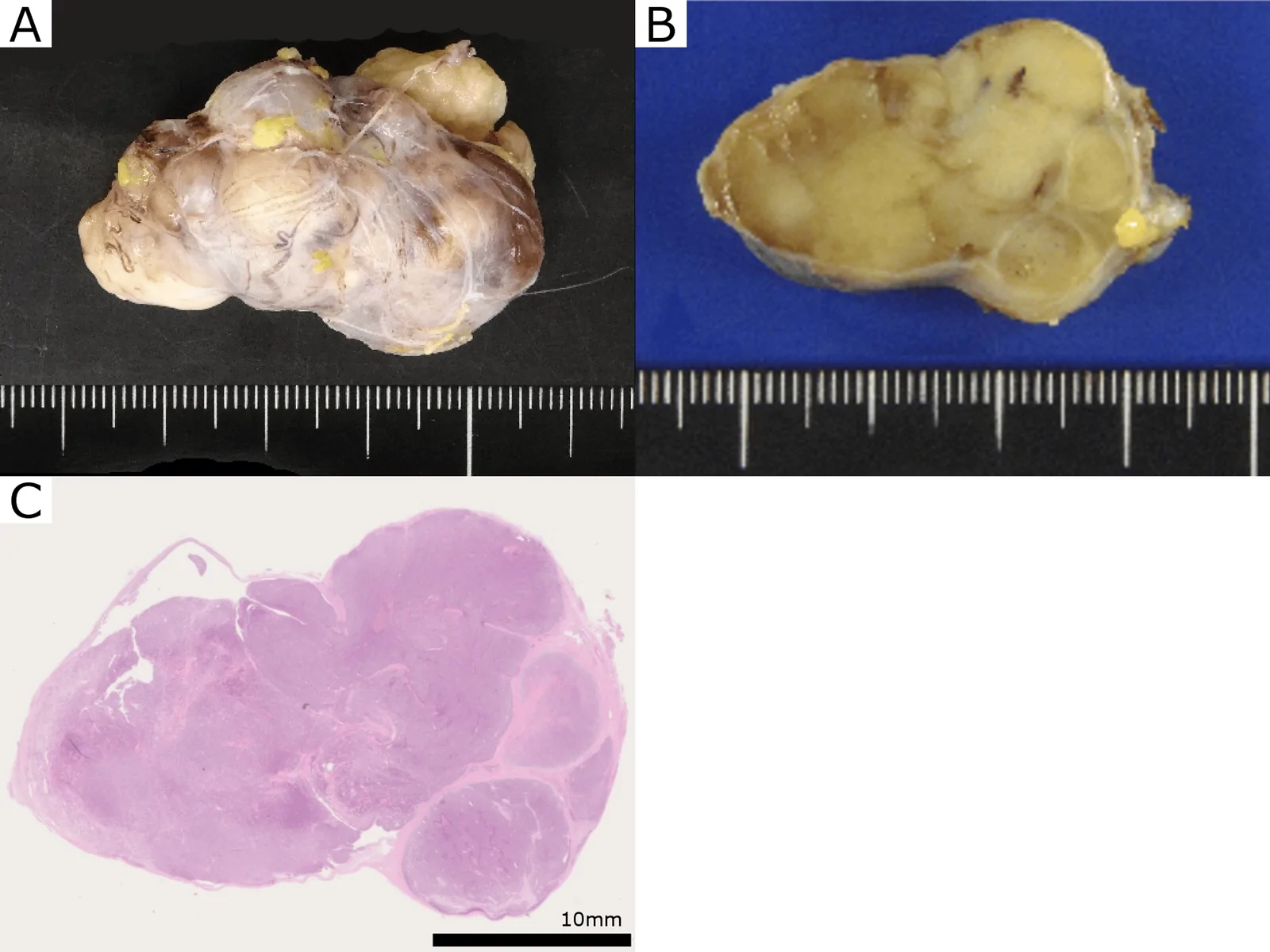
Gross and low-power findings of the resected tumor. (A) The resected specimen measured 45 × 35 × 15 mm and consisted of a well-circumscribed, yellow-tan to yellow-whitish, multinodular tumor completely surrounded by a fibrous capsule. (B) The cut surface showed a mixture of solid and semi-translucent gelatinous areas. (C) Image under view magnification shows lobulated and island-like tumor growth surrounded by a fibrous capsule (hematoxylin and eosin (H&E) staining, objective magnification ×0.4).

Gross examination of the surgical specimen revealed a 45 × 35 × 15 mm well-circumscribed, yellow-tan to yellow-whitish, multilocular nodule entirely enclosed by a capsule (Figure 3A). The cut surface revealed a mixture of solid areas and semitranslucent, gelatinous areas (Figure 3B). At low magnification, the tumor showed a lobulated or island-like growth pattern and was surrounded by a thin fibrous capsule (Figure 3C). 

**Figure 4:**
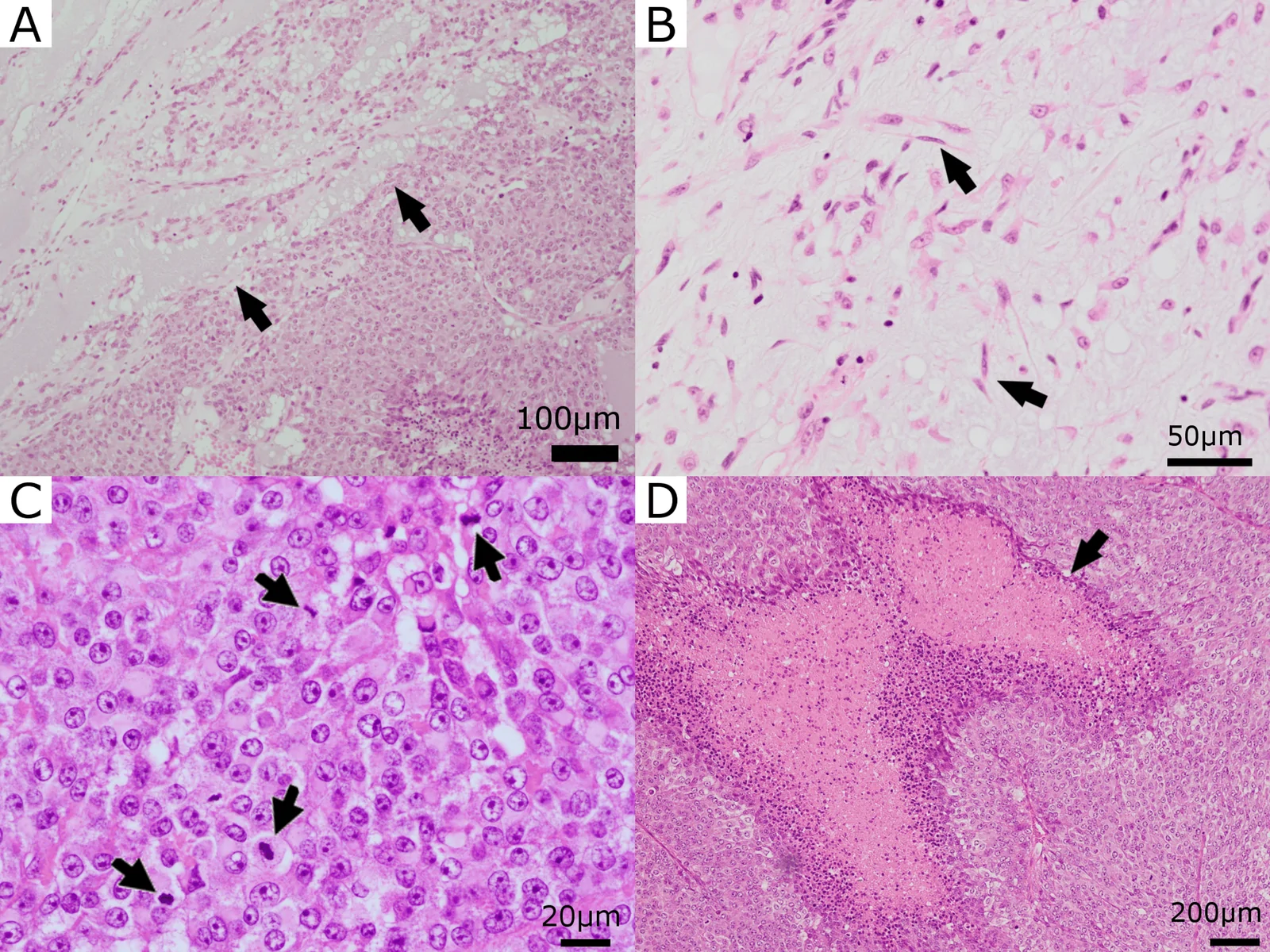
Histological findings of the resection specimen. (A) Transition between hypocellular myxoid areas (upper left) and hypercellular areas (lower right) with lobulated architecture (hematoxylin and eosin (H&E) staining, objective magnification ×4). Arrows indicate the transition zone between hypocellular myxoid and hypercellular areas. (B) Hypocellular myxoid area composed of spindle to oval tumor cells embedded in abundant myxoid stroma (arrows) (H&E, ×20). (C) Hypercellular area showing solid proliferation of round to oval tumor cells with mitotic figures (arrows) (H&E, ×60). (D) Necrotic focus within the hypercellular area (arrow) (H&E, ×20).

Transition areas between mucinous sparse and dense cellular areas, similar to those in the biopsy specimen, were observed (Figure 4A). In mucinous sparse areas, tumor cells were spindle-shaped to oval with mild nuclear atypia and were embedded in abundant myxoid stroma (Figure 4B). In dense cellular areas, the same round to oval tumor cells as those seen in the mucinous sparse areas exhibited a solid growth pattern. Furthermore, mitotic figures, averaging 9 per 10 high-power fields (×400), were present (Figure 4C), and necrotic foci were also noted in this area (Figure 4D). 

Compared with the biopsy specimen, the resection specimen demonstrated several additional histological features. Although both specimens shared transition zones between hypocellular myxoid and hypercellular areas composed of spindle-shaped to plasmacytoid tumor cells, only the resection specimen exhibited a lobulated (island-like) architecture surrounded by a thin fibrous capsule, increased mitotic activity, and focal necrosis. These additional findings substantially narrowed the differential diagnosis.

**Figure 5:**
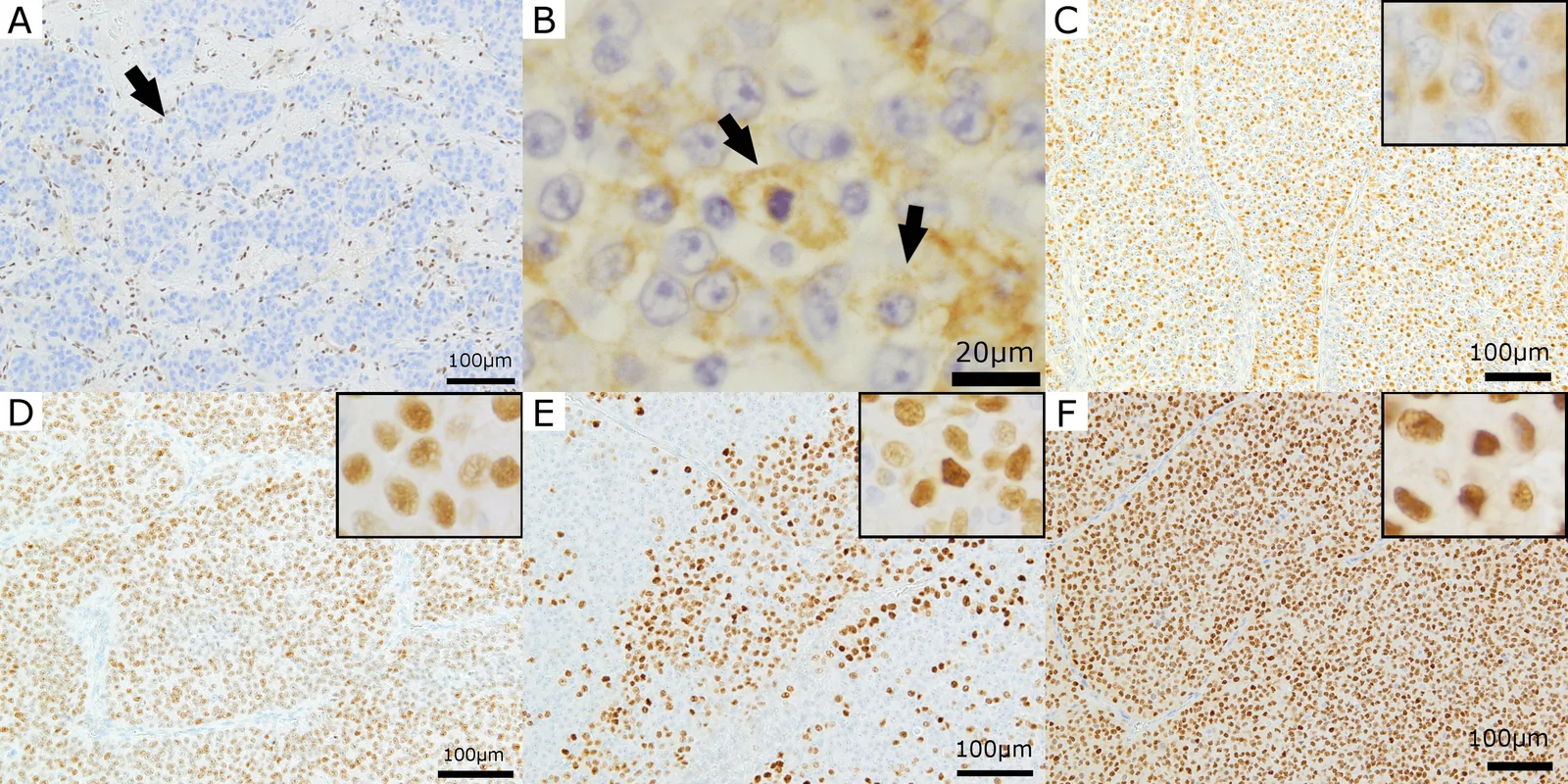
Immunohistochemical findings of the resected tumor. (A) Loss of nuclear SMARCB1 (INI1) expression in tumor cells (arrow). Retained nuclear staining in non-neoplastic stromal cells serves as an internal positive control (×10). (B) Focal positivity for epithelial membrane antigen (EMA) in tumor cells (×40). Arrows indicate EMA-positive tumor cells. (C) Strong cytoplasmic positivity for vimentin (×10). (D, E, F) Strong nuclear positivity for estrogen receptor (ER) (Figure 5D), progesterone receptor (PgR) (Figure 5E), and androgen receptor (AR) (Figure 5F) in tumor cells (all at objective magnification ×10). Insets show higher-magnification (×60) views highlighting the representative immunohistochemical staining patterns.

Immunohistochemical staining was performed using standard automated methods according to the manufacturer's protocols. Loss of SMARCB1 (INI1) expression was observed in the tumor cells (Figure 5A). Epithelial markers (AE1/AE3 and CAM5.2), myoepithelial markers (p63, alpha-smooth muscle actin (α-SMA), and glial fibrillary acidic protein (GFAP)), muscle markers (desmin, myogenin, and MyoD1), neural markers (S-100 protein, Melan-A, and Melanosome), hematolymphoid markers (CD3 and CD20), and the vascular marker CD34 were all negative. In contrast, epithelial membrane antigen (EMA) was focally positive (Figure 5B), and vimentin showed strong cytoplasmic positivity in the tumor cells (Figure 5C). In addition, the tumor cells showed strong nuclear positivity for estrogen receptor (ER), progesterone receptor (PgR), and androgen receptor (AR) (Figure 5D-5F).

**Figure 6:**
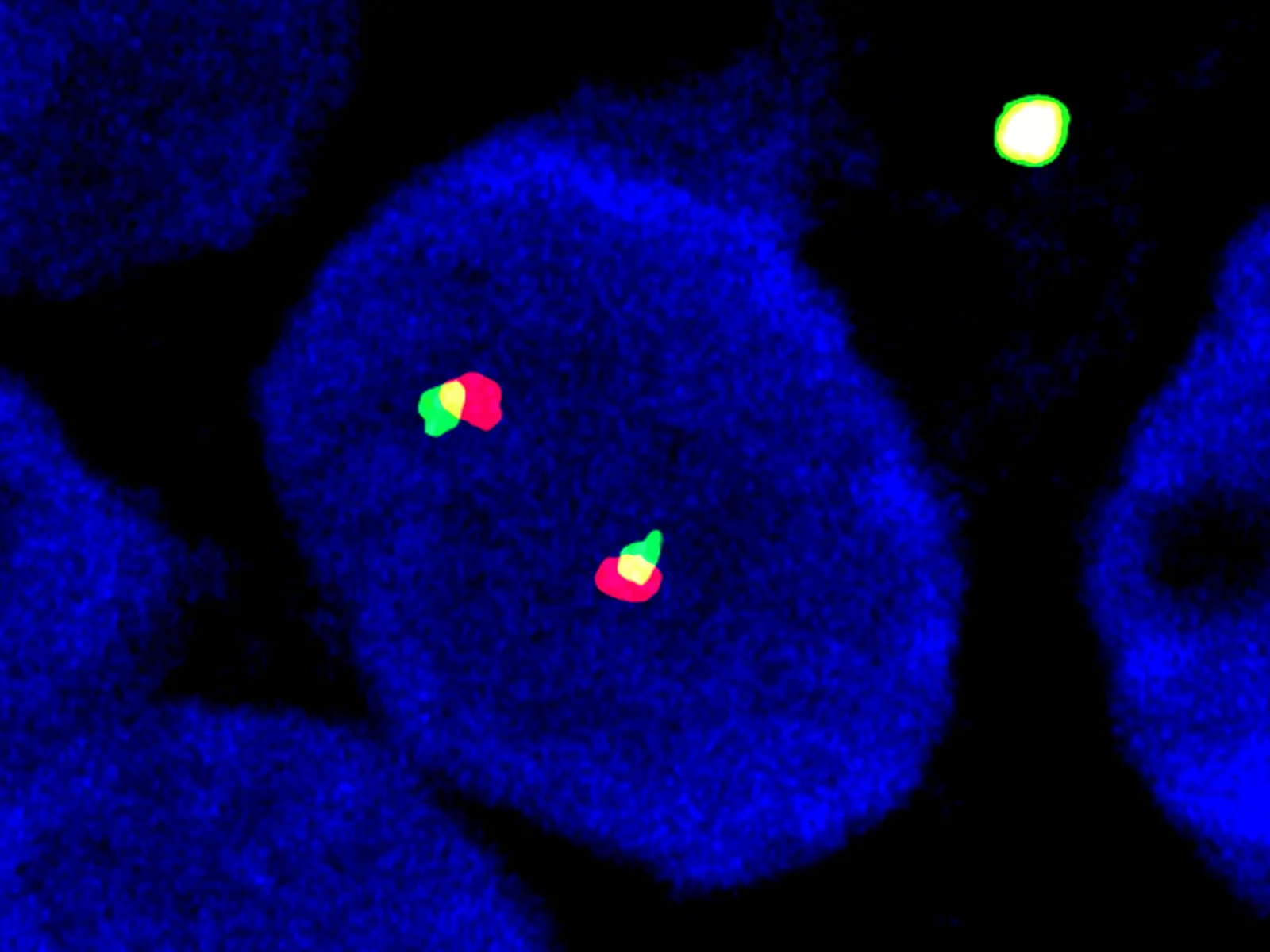
Fluorescence in situ hybridization (FISH) findings for Ewing sarcoma breakpoint region 1 (EWSR1) rearrangement. Break-apart FISH analysis performed at the Department of Pathology and Oncology, University of Occupational and Environmental Health, Japan, demonstrated no split signals, indicating absence of EWSR1 gene rearrangement.

The Ki-67 labeling index, assessed in hotspot areas at ×400 magnification, was approximately 10%. Fluorescence in situ hybridization (FISH) for Ewing sarcoma breakpoint region 1 (EWSR1) rearrangement was performed at the Department of Pathology and Oncology, University of Occupational and Environmental Health, Japan. No split signals were identified, indicating the absence of EWSR1 gene rearrangement (Figure 6).

Based on the histological, immunohistochemical, and molecular findings, the differential diagnoses included soft tissue myoepithelioma, myoepithelial carcinoma, SMARCB1-deficient sarcoma, epithelioid sarcoma, rhabdomyosarcoma, malignant melanoma, malignant lymphoma, epithelioid angiosarcoma, and other soft tissue sarcomas. Soft tissue myoepithelioma and myoepithelial carcinoma were considered because of the myxoid stroma and spindle-to-plasmacytoid morphology; however, the absence of conventional myoepithelial markers, including S-100 protein, SOX10, GFAP, α-SMA, and calponin, together with the lack of EWSR1 rearrangement, argued against these entities. Epithelioid sarcoma and other SMARCB1-deficient tumors were also considered because of the loss of SMARCB1 (INI1) expression; however, the characteristic biphasic morphology, ER/PgR positivity, and vulvar location favored MELTVR.

Based on the overall clinical, histopathological, immunohistochemical, and molecular findings, together with a review of the literature, the diagnosis of MELTVR was established. The tumor was completely excised, with no tumor cells identified at the surgical margins. No additional surgical treatment was required. At the three-month postoperative follow-up, the patient showed no evidence of recurrence and remained under regular follow-up.

## Discussion

This case highlights the diagnostic challenge of MELTVR, particularly the limitations of biopsy specimens in capturing key histological features required for definitive diagnosis. Histologically, the biopsy specimen showed spindle to plasmacytoid tumor cells with transition between hypocellular myxoid and hypercellular areas but lacked lobulated architecture, necrosis, and increased mitotic activity. In contrast, the resection specimen demonstrated characteristic diagnostic features, including lobulated architecture, increased mitotic activity, and necrosis, enabling a definitive diagnosis. This highlights that histological features associated with tumor aggressiveness, including mitotic activity and necrosis, may be underrepresented in biopsy specimens, potentially leading to underestimation of tumor aggressiveness. This discrepancy likely reflects intratumoral heterogeneity and sampling limitations associated with small biopsy specimens [1-5].

As summarized in Table 1, all previously reported MELTVR cases were diagnosed based on resection specimens. Among the 13 cases for which the full text was available, detailed histological findings from preoperative biopsy specimens were not described. Therefore, the present case provides valuable insight into the limitations of biopsy in establishing a definitive diagnosis of MELTVR by directly comparing the histological findings of the biopsy and resection specimens, emphasizing the importance of complete excision with comprehensive histopathological and immunohistochemical evaluation. Although loss of SMARCB1 expression is considered a characteristic feature of MELTVR, Aminimoghaddam et al. [4] reported an exceptional case with retained SMARCB1 expression. This case involved a large (10 cm) vulvar tumor with mild to moderate atypia but no mitotic activity or necrosis, and the tumor subsequently recurred 36 months after resection. In contrast, the present case showed SMARCB1 loss, increased mitotic activity, and focal necrosis. The significance of retained SMARCB1 expression in MELTVR remains unclear. 

**Table 1:** Clinicopathological features of previously reported cases of MELTVR and the present case. ER: estrogen receptor, PgR: progesterone receptor, EMA: epithelial membrane antigen, α-SMA: α smooth muscle actin, CK: Cytokeratin , AR: androgen receptor, +: positive, -: negative, NR: not reported

Author	Age	Clinical presentation	Tumor size	Histological findings	Immunohistochemistry		SMARCB1	Treatment	Margin	Outcome
Yoshida et al. [1]	24–70	Vulvar mass/nodule	Variable	Spindle to epithelioid cells with myxoid and hypercellular areas	ER + PgR + EMA +	variable α-SMA	Lost	Surgical excision	Variable	Mostly favorable (1-172 months)
Liu et al. [2]	43	Vulvar mass	3.5 cm	Myxoid and solid components with spindle/plasmacytoid cells			Lost	Wide local excision	NR	No recurrence
Urakami et al. [3]	54	Vulvar tumor after inguinal tumor excision	4 cm	Lobulated tumor with myxoid stroma	ER + PgR +		Lost	Surgical excision	Negative	No recurrence (10 months)
Aminimoghaddam et al. [4]	46	Subcutaneous vulvar nodules with recent enlargement and discomfort; mass involving the right labia majora and mons pubis	10 cm	Myxoid mesenchymal tissues. A mild to moderate atypia. No mitosis or necrosis		Vimentin +	Lost	Surgical resection	NR	Recurrence (36 months)
Kamboj et al. [5]	47	A mobile swelling in the subcutaneous region of right labia majora	3 cm	Solid sheets of cells with a predominant epithelioid to rhabdoid morphology and focal areas of spindling	ER + EMA +	CK -	Lost	Surgical resection	NR	No recurrence (6 months)
Present case	23	Myxoid and hypercellular areas with spindle to plasmacytoid cells, necrosis, and mitotic activity	4.5 cm	Myxoid and hypercellular areas with spindle to plasmacytoid cells, necrosis, and mitotic activity	ER + PgR + EMA +	AR +	Lost	Surgical excision	Negative	No recurrence (3 months)

In addition to its characteristic morphology and SMARCB1 deficiency [1-3,5-8], MELTVR frequently expresses hormone receptors such as ER and PgR [1-5,7,8]. Strong AR expression was also observed in the present case (Figure 5F). While ER and PgR expression has been described in most reported MELTVR cases, recent evidence indicates that ER expression is not invariable [10]. In contrast, the clinicopathological significance of AR expression remains largely unexplored [1,11]. The concurrent expression of ER, PgR, and AR in our case may support a hormone receptor-associated phenotype; however, additional studies are required to determine whether AR expression has diagnostic or biological significance in MELTVR.

## Conclusions

This case illustrates the diagnostic challenges of MELTVR, particularly the limitations of biopsy specimens in capturing its characteristic histological features. Comprehensive evaluation of the resection specimen, together with immunohistochemical and molecular analyses, was essential for establishing the correct diagnosis. In addition, the strong expression of AR observed in the present case may broaden the immunophenotypic profile of MELTVR. However, this observation is based on a single case and requires confirmation in larger case series before its diagnostic or biological significance can be established. Although no recurrence has been observed during the current three-month follow-up period, longer-term follow-up is required to better define the clinical behavior and prognosis of MELTVR.
